# Supplementary material for: A major 6 Mb superlocus is involved in pyrethroid resistance in the common bed bug Cimex lectularius
Source: Evol Appl. 2023 Apr 25;16(5):1012–28. doi: 10.1111/eva.13550 (PMC10197226; doi:10.1111/eva.13550)
Supplement: Supplementary file 1 — Data S1: [file EVA-16-1012-s001.pdf]

6 | SUPPLEMENTARY

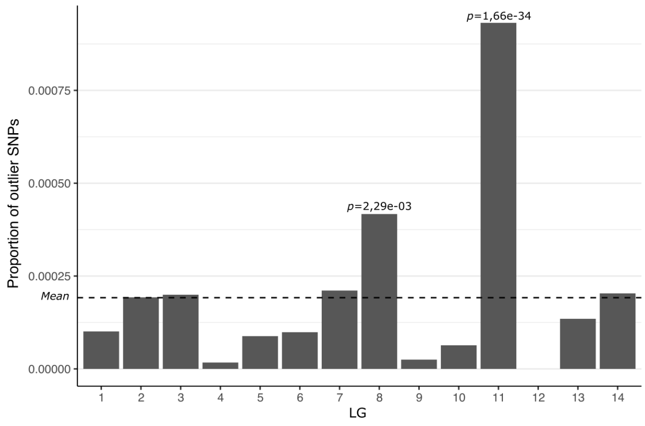

SUPPLEMENTARY FIGURE 1 Proportions of outlier SNPs among SNPs in linkage groups (LG). The dashed line represents average proportion (a = 0.00019). Binomial test were performed between observed proportion and average proportion in each LG, and significant corrected p-values were detailed above corresponding LG.

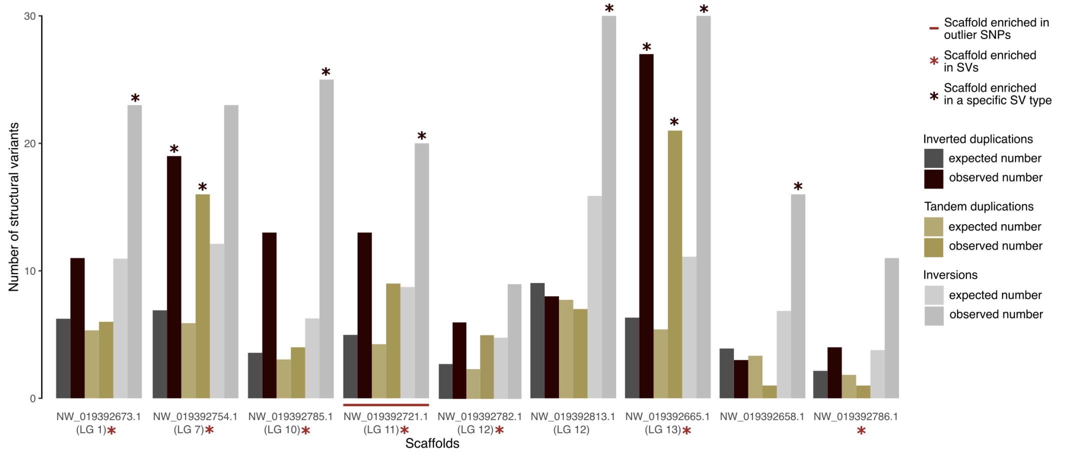

SUPPLEMENTARY FIGURE 2 Expected and observed numbers of structural variants (SVs) for scaffolds enriched with structural variants in general (delineated with a red star below) or enriched with a specific type of structural variant (namely tandem duplications, inverted duplications and inversions, delineated with a black star above corresponding observed number). The scaffold also enriched with outlier SNPs is underlined in red.

	n	slope	df	X^2	LD_{95}	95% mortality CI95
London Lab	280	1.840	27	178.10	1.3	91.2-98.8%
German Lab	200	3.123	19	172.97	0.4	89.8-100%
London Field	170	1.030	17	75.76	21.0	89.7-100%
Sweden Field	210	0.941	21	97.04	69.2	90.5-99.5%

SUPPLEMENTARY TABLE 2 Additional information for dose-mortality analysis performed on *Cimex lectularius* strains. Number of insects (n), slope, degree of freedom (df) and chi-square (X^2, computed as null deviance minus residual deviance), lethal dose (in ng) for 95% of insects in the population (LD_{95}) were given for each strains. The confidence interval at 95% (CI95) for 95% mortality was also computed for each strain using the predict function from the R/ecotox package.

Scaff.	Gene ID	Position	Annotation	Location	F_{alt} LL	F_{alt} LF	F_{ST} (pval)
2763	106666926	419008	*GST1-1*-like	exon NS	0.20	0.38	-0.01 (0.38)
	106666926	419242	*GST1-1*-like	exon NS	0.08	0.27	0.05 (0.25)
	106666926	419369	*GST1-1*-like	exon NS	0.06	0.24	0.03 (0.28)
	106663963	503362	*GST*-like	exon NS	0.10	0.46	0.16 (0.10)
2721	106663983	1833078	*CYP6B5*-like	exon NS	0.50	0.93	0.29 (0.03*)
	106663983	1833420	*CYP6B5*-like	exon NS	0.41	0.79	0.17 (0.09)
	106663983	1833792	*CYP6B5*-like	exon NS	0.46	0.78	0.11 (0.15)
	106663983	1833793	*CYP6B5*-like	exon NS	0.46	0.76	0.09 (0.18)
	106663983	1833810	*CYP6B5*-like	exon NS	0.50	0.77	0.07 (0.20)
	106663983	1833837	*CYP6B5*-like	exon NS	0.56	0.75	-0.02 (0.40)
	106663983	1836843	*CYP6B5*-like	exon NS	0.58	1,00	0.33 (0.02*)
	106663983	1838158	*CYP6B5*-like	exon NS	0.55	0.85	0.13 (0.12)
	106663982	1849007	*CYP6B1*-like	exon NS	0.27	0.72	0.28 (0.04*)
	106663982	1849044	*CYP6B1*-like	exon NS	0.29	0.75	0.2 (0.03*)
	106663982	1849067	*CYP6B1*-like	exon NS	0.29	0.63	0.15 (0.11)
	106663982	1849919	*CYP6B1*-like	exon NS	0.59	0.84	0.07 (0.21)
	106663981	1873544	*CYP6B1*-like	exon NS	0.33	0.50	0.00 (0.35)
	106663981	1873558	*CYP6B1*-like	exon NS	0.33	0.53	0.02 (0.30)
	106663981	1874026	*CYP6B1*-like	exon NS	0.59	0.66	-0.05 (0.50)
	106663981	1874033	*CYP6B1*-like	exon NS	0.54	0.57	-0.06 (0.53)
	106663981	1875827	*CYP6B1*-like	exon NS	0.07	0.25	0.01 (0.32)
	106663981	1875904	*CYP6B1*-like	exon NS	0.23	0.35	-0.04 (0.46)
	106663981	1875976	*CYP6B1*-like	exon NS	0.72	0.75	-0.09 (0.69)

SUPPLEMENTARY TABLE 3 Non-synonymous (NS) SNPs falling within putative resistant genes inside the superlocus detected, with frequency for derived allele from ancestral susceptible population (F_{alt}) superior in London Field (London Field) than in London Lab (London Lab). Gene annotation *GST* stands for glutathione-S-transferase, *CYP* for cytochromes P450. To obtain scaffold name, one should add "NW_01939" at the beginning, and ".1" at the end.

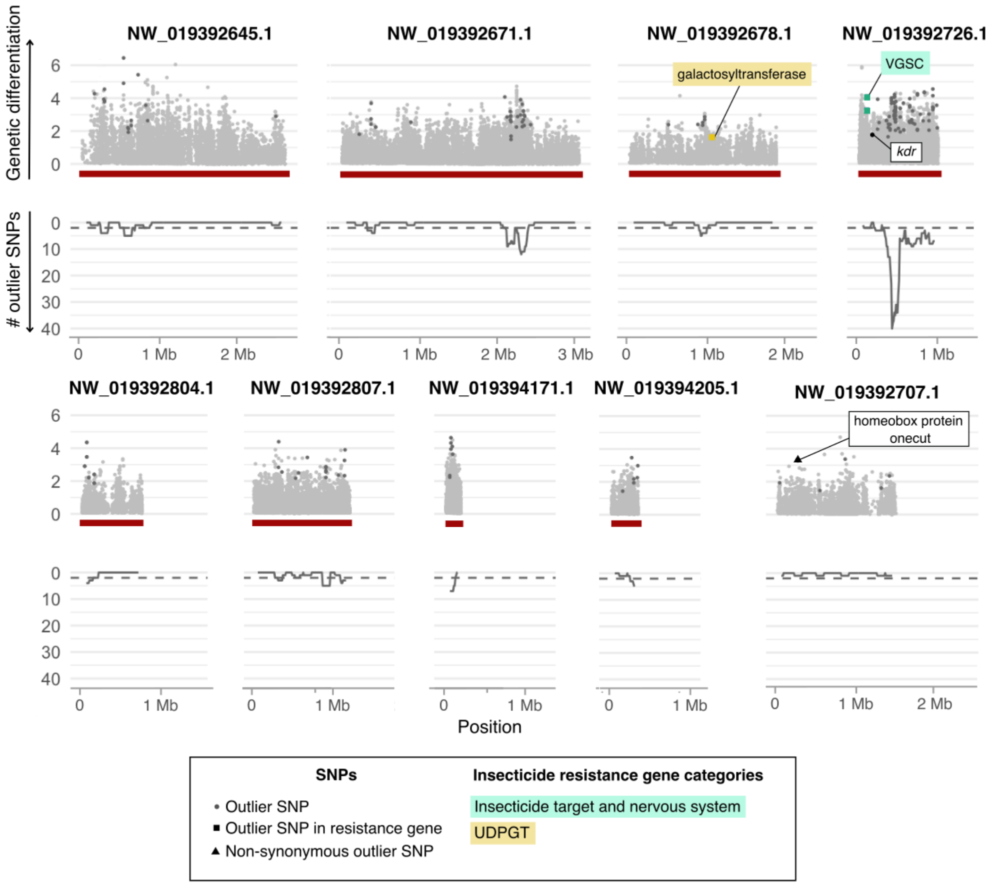

SUPPLEMENTARY FIGURE 3 Several supplementary scaffolds for *Cimex lectularius* genome not located on linkage groups. Scaffolds detected as significantly enriched in outlier SNPs are underlined in red. The scaffold carrying the unique outlier non-synonymous SNP is also represented (NW_019392707.1). In the upper part of a plot, the empirical p-value (p) of genetic differentiation index (F_{ST}) between London Lab and London Field populations in -log10 scale is plotted. The x-axis indicates the position within each scaffold. Outlier SNPs (see Materials & methods) are distinguished by a darker grey. Square-shaped points are the outlier SNPs that fall within candidate resistance genes, and are colored according to those genes' categories. Their protein products are labeled. Non-synonymous outlier mutation was represented with a black triangle. Although not an outlier SNP, the location of the non-synonymous *kdr* L925I mutation was given as an indication. Positions of genes with an expected amplified copy number are displayed with a dashed black vertical line and labelled likewise. In the lower part, "# outlier SNPs" represents the number of outlier SNPs counted in each 100 kb sliding window with a step of 10 kb. Dashed line stands for a threshold of 2 outlier SNPs, which delineates quantile 99%.

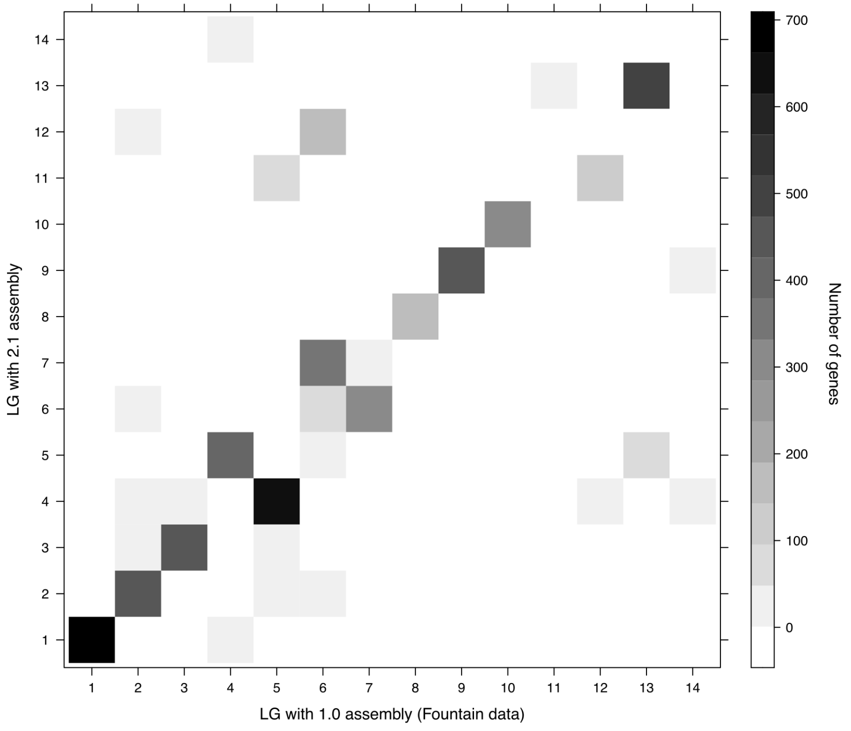

SUPPLEMENTARY FIGURE 4 Joint-distribution of genes in Linkage Groups (LG) in Fountain et al. (2016) paper and the present paper. Genes present on the first diagonal are located on the same LG in both cases.

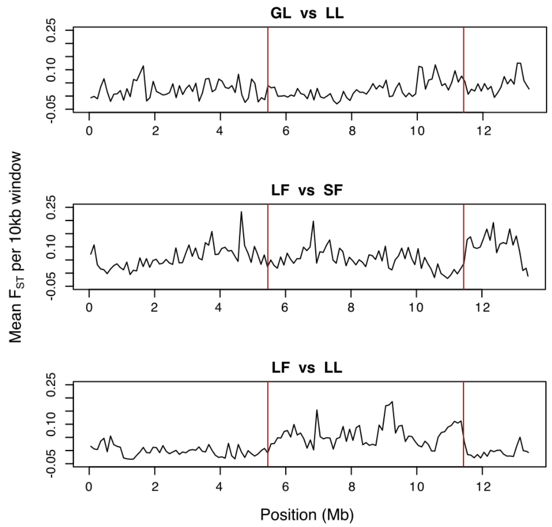

SUPPLEMENTARY FIGURE 5 Differentiation between strains over LG 11. Mean F_{ST} values were computed in 10 kb windows with 10 kb step for three comparisons: between the two susceptible strains (GL = German Lab and LL = London Lab), the two resistant strains (LF = London Field and SF = Sweden Field), and between Londonian strains (LF and LL). The position of a "superlocus" (NW_019392721.1, NW_019392763.1 and NW_019942502.1) is delineated with red lines.

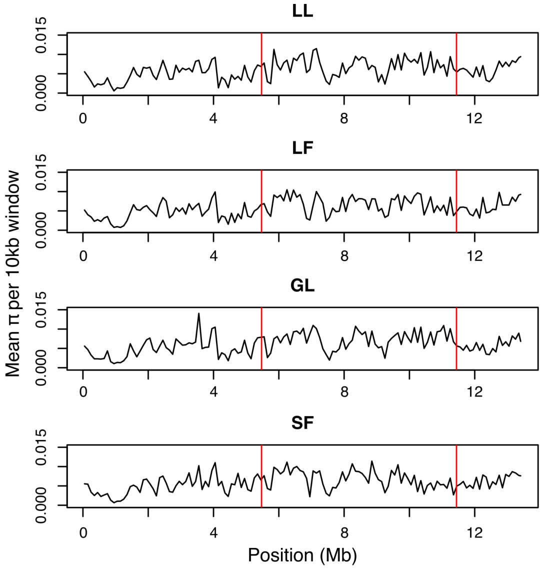

SUPPLEMENTARY FIGURE 6 Nucleotidic diversity within strains over LG 11. Mean π values were computed in 10 kb windows with 10 kb step for the four strains: two susceptible (GL = German Lab and LL = London Lab) and two resistant (LF = London Field and SF = Sweden Field). The position of a "superlocus" (NW_019392721.1, NW_019392763.1 and NW_019942502.1) is delineated with red lines.

LG	Scaff.	Event	Size	Gene ID	Annotation	F LL	F LF	F_{ST}	RDR (q)
1	2640	Inv	324 kb	106672175	sulfotransferase	0	0.09	0.05 (7%)	1.23 (1.21-1.24)
1	2679	InvDup	1.494 Mb	106661337	sulfotransferase	0.06	0.24	0.07 (4%)	1.23 (1.23)
				106661366	choline kinase				
				106661634	CYP450 306a1				
				106661588	CYP450 18a1				
1	2641	Inv	262 kb	106669934	SOD [Cu-Zn]	0	0.10	0.05 (6%)	1.22 (1.21-1.24)
				106669936	SOD [Cu-Zn] 2				
2	2979	Inv	7 kb	106665265	endocuticle str. prot.	0	0.12	0.06 (4%)	1.15 (1.15-1.29)
				106665194	endocuticle str. prot.				
13	2685	Inv	76 kb	106662993	cuticle protein	0.16	0.41	0.08 (2%)	1.23 (1.20-1.25)
		Inv	72 kb	106662993	cuticle protein	0	0.09	0.05 (7%)	1.24 (1.20-1.25)
-	2647	Inv	484 kb	106668191	NADPH oxidored.	0.06	0.26	0.08 (3%)	1.23 (1.23)
-	2656	InvDup	381 kb	106669691	glucosyltransf.	0.22	0.47	0.06 (4%)	1.24 (1.23)
-		Inv	221 kb	112126170	galactosyltransf.	0	0.24	0.13 (1%)	1.22 (1.21-1.24)
				106669680	glucuronosyltransf.				
-	2881	InvDup	27 kb	106665141	cuticle protein	0.02	0.16	0.06 (5%)	1.29 (1.26)
				106664994	cuticle protein				
				106664995	cuticle protein				
				106664992	cuticle protein				

SUPPLEMENTARY TABLE 4 Resistance genes overlapping with structural variants with extreme F_{ST} value (top 10%, specified in brackets). SVs events are delineated as tandem duplications (TandDup), inverted duplications (InvDup), and inversions (Inv). Read Depth Ratio (RDR) between London Field and London Lab was given for each event, along with the threshold quantile (q). Since duplications were all detected in higher frequency in London Field, we only showed the q75 quantile used. For inversions, we showed the q25-q75 interval. "SOD" stands for superoxide dismutase. Gene annotations have been shortened, but full informations can be found using gene ID. To obtain scaffold name, one should add "NW_01939" at the beginning, and ".1" at the end.
